# The diagnostic value and optimal cutoff of basal luteinizing hormone for central precocious puberty in premenarcheal girls stratified by weight status

**DOI:** 10.3389/fendo.2026.1900964

**Published:** 2026-07-23

**Authors:** Xin Yuan, Jing Zhang, Ying Zhang, Wenyong Wu, Ruimin Chen

**Affiliations:** 1 Department of Pediatric Endocrinology, Genetics and Metabolism, Fuzhou First General Hospital Affiliated with Fujian Medical University, Fuzhou Children’s Hospital of Fujian, Fuzhou, China; 2 Department of Pediatrics Clinical Medicine, Quanzhou Medical College, Quanzhou, China

**Keywords:** basal luteinizing hormone, BMI, central precocious puberty, diagnosis, obesity

## Abstract

**Objective:**

To investigate the diagnostic value and explore optimal cutoff value of the basal luteinizing hormone (LH) level for central precocious puberty (CPP) in premenarcheal girls with different weight status, so as to provide preliminary stratified reference thresholds and a basis for reducing unnecessary gonadotropin-releasing hormone (GnRH) stimulation tests in clinical screening.

**Methods:**

This retrospective single-center study involved girls with breast development. They were divided into groups of normal weight (n=982), overweight (n=381), obesity (n=195) and underweight (n=176) based on body mass index (BMI) z scores. All individuals underwent GnRH stimulation test. General characteristics and hormonal profiles were compared among different weight groups, and receiver operating characteristic (ROC) curves were plotted.

**Results:**

In each weight group, CPP girls had significantly higher basal LH, bone age, and uterine/ovarian volumes than non-CPP girls, and basal LH showed moderate positive correlations with peak LH (Spearman’s rho ranged from 0.557 to 0.633, all P<0.05). Basal LH had a higher area under the curve (AUC) for CPP diagnosis than basal FSH and LH/FSH ratio across all groups, with the AUC values of basal LH being 0.770 (normal weight), 0.822 (overweight), 0.749 (obesity), and 0.767 (underweight). The optimal basal LH cutoffs derived from the maximum Youden index were 0.165 IU/L (normal weight), 0.145 IU/L (overweight), 0.135 IU/L (obesity) and 0.135 IU/L (underweight), with the corresponding Youden indices of 0.402, 0.525, 0.416, and 0.456, respectively. At these thresholds, the sensitivity of basal LH for CPP diagnosis ranged from 45.8% to 84.4% and specificity ranged from 63.6% to 70.4% across all subgroups.

**Conclusions:**

Basal LH serves as a convenient indicator with moderate diagnostic discrimination for CPP screening in premenarcheal girls with different weight statuses, and weight-stratified optimal cutoffs provide individualized screening references for girls with different weight statuses and help mitigate diagnostic bias caused by body weight confounding.

## 1 Introduction

In recent years, the global incidence of central precocious puberty (CPP) in girls has increased remarkably. Over the past two decades, the age of pubertal onset has been decreasing (1), which is closely linked to the growing prevalence of childhood obesity and changes in lifestyle (2). CPP can lead to adverse outcomes including early menarche, premature epiphyseal closure with consequent reduction in final adult height, and psychosocial complications such as anxiety, depression, and body image disturbance, thus attracting widespread public attention (3). Premature thelarche (PT), a form of incomplete precocious puberty, is characterized solely by isolated breast development without other signs of sexual maturation (e.g., pubic hair growth, accelerated growth spurt) and typically resolves spontaneously (4). However, distinguishing CPP from PT in clinical practice remains challenging. Timely and accurate diagnosis of CPP is therefore crucial to initiate appropriate interventions and optimize long-term outcomes.

The gonadotropin-releasing hormone (GnRH) stimulation test is universally recognized as the gold standard for CPP diagnosis. Nevertheless, this test has notable limitations: it is time-consuming (requiring 2–3 hours of monitoring), invasive (involving multiple blood draws), and costly. Furthermore, intravenous administration of gonadorelin may induce adverse reactions such as cyanosis, hypotension, and in rare cases, anaphylactic shock (4). The European guidelines for the application of GnRH analogs recommend restricting the use of the GnRH stimulation test, particularly in girls with mild or ambiguous clinical manifestations (5). Similarly, the Chinese expert consensus emphasizes the urgent need to explore simple, safe, and non-invasive diagnostic indicators to reduce unnecessary GnRH stimulation tests (6).

Clinical guidelines and relevant studies have long recognized basal luteinizing hormone (LH) as a potential marker for the GnRH stimulation test, as it can preliminarily reflect the activation status of the hypothalamic-pituitary-gonad (HPG) axis in prepubertal girls (5–9). However, the optimal cutoff values of basal LH reported in existing studies vary widely (ranging from 0.1 to 1.5 IU/L), which severely limits the standardized clinical application of basal LH (10, 11).

The pathogenesis of CPP involves multiple factors, including genetics, nutrition, and environmental endocrine disruptors. Increased body fat is increasingly recognized as one of the key drivers of earlier puberty onset (12, 13). Previous research by Fu et al. demonstrated that obese girls with CPP had significantly lower peak LH levels than their normal-weight counterparts, and peak LH levels were negatively correlated with body mass index (BMI) (14). Our prior study further confirmed an association between BMI and basal LH levels in girls with CPP, with distinct patterns observed across different weight groups (15). Notably, BMI may be a key factor leading to the inconsistent cutoff values of basal LH in previous studies. Excess adipose tissue alters peripheral estrogen conversion and insulin metabolism, while undernutrition suppresses central kisspeptin signaling; both pathways modulate basal LH secretion independent of chronological age and breast Tanner stage. Such weight-related hormonal changes may shift the optimal diagnostic threshold of basal LH, which provides a biologically plausible hypothesis for the inconsistent cutoff values reported in previous studies. However, this hypothesis needs further mechanistic verification. Despite these findings, few studies have stratified CPP diagnostic analysis by four weight categories (underweight, normal weight, overweight, and obesity), and the underweight subgroup remains understudied.

Despite these findings, standardized weight-stratified basal LH cutoffs are still lacking. Therefore, this study aimed to evaluate the diagnostic efficacy of basal LH for CPP in premenarcheal girls stratified by BMI-Z and to establish optimal subgroup-specific cutoff values, providing a simple, non-invasive reference to reduce unnecessary GnRH stimulation testing.

## 2 Patients and methods

### 2.1 Study population

This was a retrospective cohort study conducted at a single center. We consecutively enrolled 1734 girls with early breast development (Tanner breast stage ≥2) before 8 years of age who were admitted to Fuzhou Children’s Hospital of Fujian Medical University between January 2018 and April 2024. The exclusion criteria were as follows: 1) Girls diagnosed with CPP due to organic pathology; 2) CPP girls who had experienced menarche; and 3) Girls with a basal LH level ≥ 5 IU/L. These patients presented definite HPG axis activation and could be clinically diagnosed as CPP without GnRH stimulation test; inclusion would artificially overestimate the diagnostic AUC of basal LH for ambiguous screening population. *A priori* sample size calculation was not conducted, as this study retrospectively enrolled consecutive eligible patients during the study period. Variables with missing values <5% were handled by listwise deletion to avoid statistical bias. Variables with missing values were bone age and pelvic ultrasound volumetric parameters, with missing rates both less than 3%. Fifteen patients lacked bone age measurements, and 17 lacked complete uterine and ovarian volume data. After listwise deletion for incomplete records, 21 patients were excluded from final analysis to avoid statistical bias.

The study protocol was approved by the Medical Ethics Committee of Fuzhou Children’s Hospital of Fujian Medical University (Approval No. 201927), and was carried out in compliance with the Declaration of Helsinki. Written informed consent was obtained from a parent or guardian on behalf of all participants.

### 2.2 Clinical assessment

Height and weight were measured by trained nurses. BMI-Z were calculated based on the age- and sex-specific reference values of Li Hui et al. for Chinese children and adolescents (16). Secondary sexual examination was conducted by trained pediatric endocrinologists, including evaluation of breast development and distribution of pubic hair. Breast development was evaluated visually and by palpation. In overweight and obese girls, breast development was reconfirmed by ultrasound as part of routine evaluation to distinguish true breast development from adipomastia. In the girls where Tanner stage for breast development was dissimilar, the breast that was more mature was documented for evaluation purposes. Bone age (BA) was based on left hand radiograph including phalanges of fingers, carpal bones and distal radius and ulna. BA was interpreted by two independent laboratory technicians who were blinded to the patients’ clinical and laboratory data using the method of Tanner-Whitehouse 3 (TW3). Discrepancies were resolved by consensus. Ultrasound examinations were performed according to conventional full bladder technique. Longitudinal (L), anteroposterior (AP), and transverse (T) diameters of the uterus as a whole, the corpus and cervix separately, and of the ovaries were measured. The formula for a prolate ellipsoid (V = L × AP × T × 0.5233) was used to calculate the volume (V) of both the uterus and ovaries.

Baseline blood samples (0min) were taken before intravenous (IV) administration of gonadorelin (2.5 μg/kg, maximum 100 μg), and additional samples of LH and FSH were drawn at 30, 60, 90 and 120 min after the IV GnRH. LH and FSH were measured by chemiluminescence (Siemens Healthcare Diagnostics, Los Angeles, CA, USA) following the manufacturer’s calibration standards. Blood samples were centrifuged within 2 hours of collection. The lower limit of sensitivity for LH and FSH is 0.1 IU/L. The intra-assay coefficient varies from 2.6% to 8.5% whereas the inter-assay coefficient varies from 3.7% to 11.9%.

### 2.3 Diagnosis and weight classification

The diagnosis of CPP depends upon the combination of clinical manifestations and peak LH response to the GnRH stimulation test. The cutoff level for the diagnosis of CPP was peak LH ≥ 5IU/L (4).

Subjects were classified according to their BMI-Z: normal weight (BMI-Z for age between -1SD to 1SD), overweight (BMI-Z for age between 1SD to 2SD), obesity (BMI-Z for age ≥2SD), and underweight (BMI-Z for age <-1SD).

### 2.4 Statistical analysis

All analyses were performed using SPSS 26.0 (IBM Corp., Armonk, NY, USA). Normally distributed data were presented as mean ± standard deviation; non-normal data were expressed as median (P25, P75). Independent t-test and Mann-Whitney U test were used for two-group comparisons. Kruskal–Wallis test was applied for multiple-group overall comparison. Spearman correlation was used to analyze the association between basal and peak hormones. ROC curves were constructed to calculate AUC, optimal cutoff (maximum Youden index), sensitivity, and specificity. A two-tailed *P* < 0.05 was considered statistically significant.

No prospective sample size estimation was conducted for this retrospective consecutive cohort. *Post-hoc* calculation based on our ROC data indicated that future validation studies need at least 32 CPP and 20 non-CPP children per weight subgroup (α=0.05, power=0.8, minimal expected AUC = 0.749), with a 15% allowance for missing data, equivalent to a total of ≥240 subjects across four weight groups.

## 3 Results

### 3.1 Basic clinical features and hormone profiles stratified by weight status

A total of 1734 girls with early breast development were enrolled, including 1079 CPP and 655 non-CPP participants. The overall mean age was 7.56 ± 0.91 years and 7.19 ± 0.85 years for CPP and non-CPP groups, respectively.

Of the 1734 girls, 1293 were Tanner stage 2 and 441 were Tanner stage 3.Within the non-CPP group (n = 655), 551 patients presented Tanner stage 2 breast development, while the remaining 104 patients were classified as Tanner stage 3. For the CPP cohort (n = 1079), 742 girls were at Tanner stage 2 and 337 at Tanner stage 3. The percentage of Tanner stage 3 breast development was significantly elevated in the CPP group relative to the non-CPP group (χ2 = 50.671, *P* < 0.001).

Based on BMI-Z, the CPP girls (n=1079) were subdivided into normal weight (n=619), overweight (n=237), obesity (n=118), and underweight (n=105). The non-CPP girls (n=655) were subdivided into normal weight (n=363), overweight (n=144), obesity (n=77), and underweight (n=71).

Within each weight status category, there was no statistically significant difference in BMI-Z between the CPP and non-CPP groups (all *P* > 0.05). However, girls in the CPP group exhibited significantly higher chronological age, BA, basal LH level, basal FSH level, basal LH/FSH ratio, peak LH level, peak FSH level, peak LH/FSH ratio, as well as uterine and ovarian volumes, compared with their non-CPP counterparts (all *P* < 0.05). Statistically significant between-group differences of BMI-Z were found in obesity (*P* = 0.021) and overweight subgroups (*P* = 0.039), whereas no significant BMI-Z differences were detected in normal weight and underweight subgroups (both *P* > 0.05). In contrast, chronological age, BA, basal and peak gonadotropins, and uterine/ovarian volumes were significantly higher in the CPP group (all *P* < 0.05) (**Tables 1**–**3**).

**Table 1:** Clinical and laboratory characteristics of the subjects.

Index	CPP	Non-CPP	T/Z value	P value
n	1079	655		
Age(years)	7.56 ± 0.91	7.19 ± 0.85	8.466	< 0.001
BA(years)	9.59 ± 1.57	8.75 ± 1.47	11.087	< 0.001
Height(cm)	127.94 ± 7.75	125.99 ± 7.70	5.112	< 0.001
Weight(kg)	26.82 ± 5.77	25.98 ± 5.78	2.961	0.003
BMI(kg/m2)	16.23 ± 2.23	16.21 ± 2.44	0.155	0.877
BMI-Z	0.47 ± 1.18	0.49 ± 1.27	-0.422	0.673
Basal LH (IU/L)	0.27 (0.15, 0.72)	0.12 (0.10, 0.18)	-19.623	<0.001
Basal FSH (IU/L)	2.66 (1.86, 4.04)	1.79 (1.26, 2.45)	-15.225	<0.001
Basal LH/FSH	0.11 (0.07, 0.22)	0.08 (0.06, 0.12)	-10.503	<0.001
Peak LH (IU/L)	9.75 (6.96, 16.80)	3.60 (2.87, 4.28)	-34.950	<0.001
Peak FSH (IU/L)	13.20 (10.29, 17.40)	10.30 (7.69, 13.40)	-12.555	<0.001
Peak LH/FSH	0.81 (0.51, 1.38)	0.33 (0.25, 0.44)	-26.598	<0.001
Uterine size (cm3)	1.41 (1.09, 1.88)	1.10 (0.91, 1.38)	-12.547	<0.001
Left ovary size (cm3)	1.86 (1.35, 2.44)	1.56 (1.19, 2.14)	-6.315	<0.001
Right ovary size (cm3)	1.87 (1.33, 2.46)	1.56 (1.23, 2.09)	-6.784	<0.001

BA, bone age; CPP, central precocious puberty; LH, luteinizing hormone; FSH, follicle-stimulating hormone; AUC, area under the curve.

**Table 2:** Clinical characteristics of girls with CPP and non-CPP girls stratified by BMI-Z status.

Group	Subgroup	n	Age(years)	BA(years)	Height(cm)	Weight(kg)	BMI(kg/m²)	BMI-Z
obesity	CPP	118	7.62 ± 1.01	10.22 ± 1.46	131.39 ± 8.19	35.71 ± 5.88	20.54 ± 1.49	2.48 ± 0.42
	Non-CPP	77	7.27 ± 0.93	9.45 ± 1.31	128.98 ± 6.63	34.89 ± 5.72	20.87 ± 2.28	2.65 ± 0.63
	T value		2.366	3.736	2.164	0.961	-1.212	-2.330
	P value		0.019	<0.001	0.032	0.338	0.227	0.021
overweight	CPP	237	7.67 ± 0.88	9.98 ± 1.41	130.13 ± 7.23	30.45 ± 4.04	17.89 ± 0.80	1.44 ± 0.30
	Non-CPP	144	7.27 ± 0.91	9.17 ± 1.44	127.88 ± 7.72	29.36 ± 4.26	17.84 ± 0.75	1.50 ± 0.29
	T value		4.243	5.359	2.872	2.516	0.632	-2.076
	P value		<0.001	<0.001	0.004	0.012	0.528	0.039
Normal weight	CPP	619	7.56 ± 0.91	9.59 ± 1.57	127.94 ± 7.75	26.82 ± 5.77	16.23 ± 2.23	0.47 ± 1.18
	Non-CPP	363	7.19 ± 0.85	8.75 ± 1.47	125.99 ± 7.70	25.98 ± 5.78	16.21 ± 2.44	0.49 ± 1.27
	T value		8.466	11.087	5.112	2.961	0.155	-0.422
	P value		<0.001	<0.001	<0.001	0.003	0.877	0.673
Underweight	CPP	105	7.27 ± 1.01	8.66 ± 1.79	123.54 ± 8.82	20.17 ± 3.10	13.14 ± 0.55	-1.53 ± 0.51
	Non-CPP	71	7.08 ± 0.86	8.15 ± 1.47	122.69 ± 8.37	19.82 ± 2.75	13.11 ± 0.56	-1.54 ± 0.53
	T value		1.248	1.984	0.637	0.760	0.350	0.159
	P value		0.214	0.049	0.525	0.448	0.727	0.874

BA, bone age; CPP, central precocious puberty; LH, luteinizing hormone; FSH, follicle-stimulating hormone; AUC, area under the curve.

**Table 3:** Auxiliary findings of girls with CPP and non-CPP girls stratified by BMI-Z.

Group	Subgroup	n	Basal LH (IU/L)	Basal FSH (IU/L)	Basal LH/FSH	Peak LH (IU/L)	Peak FSH (IU/L)	Peak LH/FSH	Uterine size (cm³)	Left ovary size (cm³)	Right ovary size (cm³)
obesity	tpdeCPP	118	0.27 (0.14, 1.07)	2.61 (1.62, 4.15)	0.11 (0.08, 0.29)	9.16 (6.96, 16.83)	12.85 (9.79, 17.00)	0.85 (0.53, 1.38)	1.42 (1.11, 1.96)	1.95 (1.39, 2.64)	1.94 (1.39, 2.57)
	Non-CPP	77	0.12 (0.10, 0.19)	1.58 (1.02, 2.70)	0.10 (0.07, 0.12)	3.12 (2.25, 4.02)	9.60 (7.65, 12.95)	0.28 (0.23, 0.39)	1.13 (0.87, 1.48)	1.40 (1.12, 2.04)	1.44 (1.24, 2.01)
	Z value		-5.950	-4.530	-3.608	-11.764	-4.113	-9.478	-4.498	-3.636	-3.602
	P value		<0.001	<0.001	<0.001	<0.001	<0.001	<0.001	<0.001	<0.001	<0.001
overweight	CPP	237	0.34 (0.17, 1.11)	2.92 (2.06, 4.40)	0.12 (0.08, 0.26)	10.50 (7.25, 17.80)	12.90 (10.35, 17.00)	0.88 (0.54, 1.47)	1.50 (1.13, 2.15)	2.04 (1.44, 2.53)	2.16 (1.50, 2.56)
	Non-CPP	144	0.12 (0.10, 0.18)	1.78 (1.23, 2.41)	0.08 (0.05, 0.14)	3.56 (2.79, 4.29)	10.60 (7.92, 14.56)	0.32 (0.25, 0.43)	1.14 (0.93, 1.45)	1.57 (1.18, 2.04)	1.52 (1.25, 2.09)
	Z value		-10.584	-8.441	-5.922	-16.371	-4.920	-13.114	-6.090	-4.969	-5.097
	P value		<0.001	<0.001	<0.001	<0.001	<0.001	<0.001	<0.001	<0.001	<0.001
Normal weight	CPP	619	0.27 (0.15, 0.63)	2.65 (1.84, 3.96)	0.11 (0.07, 0.21)	9.65 (6.95, 17.40)	13.10 (10.20, 17.10)	0.80 (0.54, 1.42)	1.41 (1.06, 1.85)	1.79 (1.33, 2.38)	1.80 (1.32, 2.42)
	Non-CPP	363	0.12 (0.10, 0.19)	1.83 (1.31, 2.43)	0.08 (0.06, 0.12)	3.62 (3.01, 4.27)	10.30 (7.69, 13.10)	0.35 (0.27, 0.46)	1.09 (0.91, 1.35)	1.56 (1.20, 2.25)	1.58 (1.24, 2.12)
	Z value		-14.221	-10.978	-7.158	-26.187	-10.014	-19.859	-9.557	-3.813	-4.144
	P value		<0.001	<0.001	<0.001	<0.001	<0.001	<0.001	<0.001	<0.001	<0.001
Underweight	CPP	105	0.21 (0.14, 0.52)	2.52 (1.87, 3.74)	0.10 (0.06, 0.17)	8.79 (6.69, 13.20)	15.00 (11.00, 20.40)	0.61 (0.44, 1.01)	1.25 (1.01, 1.62)	1.45 (1.20, 2.21)	1.56 (1.13, 2.28)
	Non-CPP	71	0.11 (0.10, 0.14)	1.72 (1.26, 2.38)	0.07 (0.05, 0.10)	3.85 (3.03, 4.36)	11.50 (7.13, 14.00)	0.35 (0.24, 0.42)	1.04 (0.90, 1.33)	1.64 (1.18, 2.20)	1.59 (1.23, 2.10)
	Z value		-6.099	-4.822	-3.298	-11.241	-4.385	-7.289	-2.943	-0.235	-1.69
	P value		<0.001	<0.001	0.001	<0.001	<0.001	<0.001	0.003	0.814	0.091

BA, bone age; CPP, central precocious puberty; LH, luteinizing hormone; FSH, follicle-stimulating hormone; AUC, area under the curve.

### 3.2 Correlation between basal and peak LH/FSH

Basal LH, FSH, and LH/FSH ratio were all positively correlated with corresponding peak levels in every weight subgroup (all *P* < 0.05), among them basal LH showed moderate positive correlations with peak LH (Spearman’s rho ranged from 0.557 to 0.633). Data are shown in **Table 4**.

**Table 4:** Correlation analysis of basic values and peak values of LH, FSH, LH/FSH in girls with different weight status.

Group	n	Index	Basal value	Peak value	Spearman’s rho	P value
Obesity	195	LH (IU/L)	0.18 (0.10, 0.42)	5.85 (3.68, 10.70)	0.557	<0.001
		FSH (IU/L)	2.16 (1.33, 3.39)	11.30 (8.64, 15.80)	0.217	0.002
		LH/FSH ratio	0.10 (0.07, 0.18)	0.53 (0.31, 0.98)	0.535	<0.001
Overweight	381	LH (IU/L)	0.21 (0.12, 0.59)	6.56 (4.13, 12.60)	0.625	<0.001
		FSH (IU/L)	2.36 (1.63, 3.85)	12.20 (8.82, 16.10)	0.261	<0.001
		LH/FSH ratio	0.11 (0.07, 0.20)	0.55 (0.34, 1.06)	0.619	<0.001
Normal weight	982	LH (IU/L)	0.19 (0.11, 0.41)	6.65 (4.09, 11.60)	0.576	<0.001
		FSH (IU/L)	2.28 (1.60, 3.31)	11.90 (9.26, 15.63)	0.360	<0.001
		LH/FSH ratio	0.10 (0.06, 0.17)	0.55 (0.36, 0.98)	0.581	<0.001
Underweight	176	LH (IU/L)	0.15 (0.10, 0.30)	6.11 (4.20, 9.99)	0.633	<0.001
		FSH (IU/L)	2.15 (1.58, 3.14)	13.45 (9.31, 17.78)	0.413	<0.001
		LH/FSH ratio	0.08 (0.06, 0.13)	0.45 (0.33, 0.70)	0.573	<0.001

BA, bone age; CPP, central precocious puberty; LH, luteinizing hormone; FSH, follicle-stimulating hormone; AUC, area under the curve.

### 3.3 Analysis of the diagnostic efficacy of basal LH, FSH, and LH/FSH ratio for CPP in girls with different weight status

**Figure 1:**
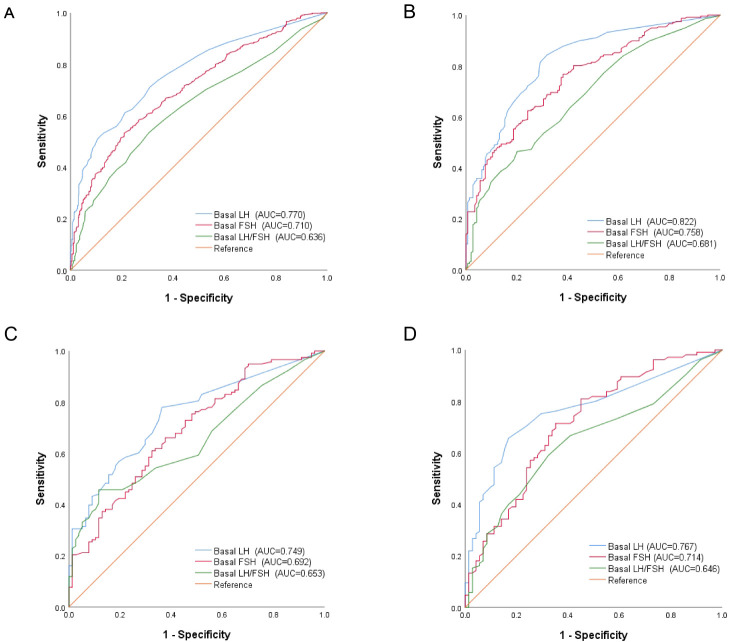
ROC curves for diagnostic efficacy of basal LH, basal FSH and basal LH/FSH ratio in girls with central precocious puberty **(A)** Normal weight group, **(B)** Overweight group, **(C)** Obesity group, **(D)** Underweight group.

ROC analyses revealed basal LH outperformed basal FSH and the LH/FSH ratio for CPP discrimination in all four weight subgroups, with AUCs of 0.770 (normal weight), 0.822 (overweight), 0.749 (obesity), and 0.767 (underweight) (all *P* < 0.001) (**Figure 1**).

The optimal basal LH cutoff value was determined according to the maximum.

Youden index: 0.165 IU/L (normal weight), 0.145 IU/L (overweight), 0.135 IU/L (obesity and underweight). Corresponding Youden indices were 0.402, 0.525, 0.416, and 0.456, with sensitivity 45.8%–84.4% and specificity 63.6%–70.4% (**Table 5**). Basal LH was superior to basal FSH and LH/FSH ratio across all subgroups.

**Table 5:** The cutoff points, sensitivity and specificity of basal LH, FSH, LH/FSH values for diagnosing CPP in girls with different weight status.

Group	Index	Optimal cutoff	AUC(SE)	95%CI	Sensitivity (%)	Specificity (%)	Youden index	P value
Normal weight	Basal LH (IU/L)	0.165	0.770(0.015)	0.741–0.799	71.1	69.1	0.402	<0.001
	Basal FSH (IU/L)	2.365	0.710(0.017)	0.677–0.742	58.5	73.3	0.318	<0.001
	LH/FSH ratio	0.105	0.636(0.018)	0.602–0.671	53.5	69.4	0.229	<0.001
Overweight	Basal LH (IU/L)	0.145	0.822(0.022)	0.779–0.865	84.4	68.1	0.525	<0.001
	Basal FSH (IU/L)	1.275	0.758(0.025)	0.710–0.806	93.2	29.9	0.231	<0.001
	LH/FSH ratio	0.065	0.681(0.028)	0.627–0.735	84.0	38.2	0.222	<0.001
Obesity	Basal LH (IU/L)	0.135	0.749(0.035)	0.681–0.817	78.0	63.6	0.416	<0.001
	Basal FSH (IU/L)	1.020	0.692(0.038)	0.617–0.767	94.9	24.7	0.196	<0.001
	LH/FSH ratio	0.145	0.653(0.039)	0.577–0.728	45.8	88.3	0.341	<0.001
Underweight	Basal LH (IU/L)	0.135	0.767(0.036)	0.696–0.837	75.2	70.4	0.456	<0.001
	Basal FSH (IU/L)	1.355	0.714(0.040)	0.637–0.792	91.4	29.6	0.210	<0.001
	LH/FSH ratio	0.075	0.646(0.042)	0.565–0.728	66.7	59.2	0.259	0.001

BA, bone age; CPP, central precocious puberty; LH, luteinizing hormone; FSH, follicle-stimulating hormone; AUC, area under the curve.

## 4 Discussion

This study, the first to include underweight girls in stratified CPP diagnostic analyses, introduces weight stratification—a well-documented modulator of pubertal LH secretion (17). By stratifying premenarcheal girls with early breast development by BMI-z, we explored the diagnostic value and weight-specific optimal cutoffs of basal LH for CPP, addressing the long-standing limitation of inconsistent universal thresholds (11) and refining the basal LH diagnostic system for girls across weight statuses.

Moderate correlation coefficients indicated that basal LH could only partially mirror GnRH-stimulated peak LH response, which further supported that basal LH merely serves as a screening marker rather than a complete replacement for the gold-standard GnRH stimulation test.

### 4.1 Diagnostic efficacy of basal LH for CPP in girls with different weight statuses

ROC curve analysis confirmed that basal LH outperformed basal FSH and the LH/FSH ratio in CPP diagnosis across all weight groups, with all AUC values significantly greater than 0.5. These AUCs are substantially higher than those reported in most unstratified studies [e.g., AUC = 0.620 (18), low diagnostic efficiency (10)], weight stratification mitigates threshold bias caused by metabolic heterogeneity and reduces misdiagnosis risk when applying a single universal LH cutoff.

The overweight group exhibited the highest AUC (0.822), consistent with Reinehr and Roth (13), who reported that mild adiposity mildly activates the pubertal HPG axis without severe obesity-induced negative feedback inhibition, may improve the separation of basal LH values between CPP and non-CPP patients. The optimal basal LH cutoffs identified—0.165 IU/L (normal weight), 0.145 IU/L (overweight), and 0.135 IU/L (obesity/underweight)—fall within the 0.1–1.5 IU/L range reported in literature (10, 11) but resolve the critical issue of universal cutoff inconsistency. For example, Ding et al. (19) proposed a universal cutoff of 0.35 IU/L (specificity=76.35%), while our weight-specific thresholds balance sensitivity (45.8%–84.4%) and specificity (63.6%–70.4%) for personalized application.

Beyond the overall diagnostic superiority, the weight-specific cutoffs exhibited distinct characteristics across subgroups. Notably, obesity and underweight groups shared the lowest cutoff (0.135 IU/L). For obese girls, this aligns with prior findings of lower basal/peak LH levels in obese CPP girls (15, 20) and Fu et al.’s (14) observation of a negative correlation between peak LH and BMI, highlighting the need for a lower threshold to avoid missed diagnoses in girls with adipomastia-related ambiguous sexual maturation (17). For underweight girls—long overlooked in CPP research—this cutoff reflects suppressed basal LH secretion due to HPG axis modulation by malnutrition (7, 11).

In normal weight girls (the largest clinical subgroup), the 0.165 IU/L cutoff achieves balanced sensitivity (71.1%) and specificity (69.1%), outperforming the universal 0.35 IU/L threshold (19) in routine screening applicability. Collectively, weight-stratified cutoffs enhance basal LH diagnostic precision, addressing the limitations of one-size-fits-all thresholds and providing a practical tool to reduce unnecessary GnRH stimulation tests—aligning with international guidelines (5, 6) for minimally invasive CPP diagnosis.

Notably, bilateral ovarian volumes showed no statistical difference between CPP and non-CPP underweight girls (left ovary *P* = 0.814, right ovary *P* = 0.091), which differed from other three weight subgroups. Chronic undernutrition restricts ovarian follicular development and reduces baseline ovarian volume universally, counterbalancing the anatomical enlargement induced by HPG axis activation in CPP patients. Accordingly, ovarian volumetric ultrasound presents limited auxiliary diagnostic value for underweight girls suspected of CPP.

The numerical differences between stratified optimal LH cutoffs were relatively narrow (0.135, 0.145 and 0.165 IU/L). However, such minor numerical variations carried definite clinical screening implications. Physiological modulation by excess adipose tissue or malnutrition downregulated baseline LH secretion in obese and underweight girls, meaning mild LH elevation under HPG axis activation could still indicate CPP and necessitate lower diagnostic thresholds.

### 4.2 Mechanisms underlying the association between weight status and basal LH cutoffs

Distinct cutoffs across weight groups reflect body mass-related modulation of the HPG axis. In obese girls, previous mechanistic literature hypothesized that obesity-induced hyperinsulinemia reduces sex hormone-binding globulin synthesis (21), while excess adipose tissue enhances aromatase activity (22), elevating systemic estrogen levels. Chronic high estrogen exposure reduces hypothalamic–pituitary–gonadal (HPG) axis responsiveness to GnRH stimulation, blunting LH secretion and necessitating a lower cutoff—supported by reduced sleep-related LH secretion in obese peripubertal girls (23) and lower GnRH-stimulated LH peaks in overweight CPP girls (20). These biological pathways remain speculative, as our study did not measure insulin, SHBG or aromatase concentrations to validate these mechanisms directly.

In underweight girls, energy deficiency suppresses hypothalamic kisspeptin expression and HPG axis activation via central metabolic pathways (24), reducing basal LH secretion—consistent with reports of nutritional deficiency-associated gonadotropin reduction (25). This explains the 0.135 IU/L cutoff, where even mild LH elevation indicates HPG axis activation amid impaired secretion. For overweight girls, mild adiposity moderately activates the HPG axis without inhibitory effects, resulting in the highest AUC and moderate cutoff (0.145 IU/L).

These findings confirm body mass as a key modifier of HPG axis function and LH secretion, reinforcing international (5) and Chinese (6) recommendations to consider nutritional status in CPP diagnosis. Weight-stratified thresholds provide evidence-based tools for clinical implementation, with personalized application improving diagnostic accuracy.

## 5 Limitations

This single-center retrospective study has multiple limitations. First, no *a priori* sample size calculation was conducted, and sample sizes were unbalanced across weight subgroups, potentially weakening the reliability of stratified LH cutoffs. Second, analyses were not adjusted for breast Tanner stage, and no multivariate logistic regression was performed. Third, DeLong’s test was not used to compare AUCs between subgroups. Fourth, the derived cutoffs are only applicable to this Siemens chemiluminescence assay and lie near the lower detection limit. Fifth, patients with basal LH ≥5 IU/L were excluded from enrollment. Finally, narrow numerical differences among thresholds create challenges for clinical interpretation. Future prospective multi-center studies stratified by both weight and Tanner stage are needed to validate these cutoffs.

## 6 Conclusion

We established exploratory weight-stratified basal LH cutoffs for girls with early breast development. Basal LH exhibited moderate screening performance superior to basal FSH and LH/FSH ratio, with the overweight subgroup showing the numerically highest AUC. Obese and underweight girls required a lower cutoff (0.135 IU/L) due to suppressed HPG axis activity. Weight-stratified thresholds mitigate bias from universal single standards, yet their incremental diagnostic benefit lacks evidence from model comparison and external validation. Basal LH is merely a screening marker and cannot substitute GnRH stimulation testing; these stratified cutoffs need prospective confirmation before clinical adoption.

## Data Availability

The original contributions presented in the study are included in the article/supplementary material. Further inquiries can be directed to the corresponding author.
